# Distinct Risk, Rescue, and Psychopathology Profiles of Non-Suicidal Self-Injury Compared to Suicide Attempts in Patients Presenting to the Emergency Department

**DOI:** 10.1192/j.eurpsy.2026.12073

**Published:** 2026-07-31

**Authors:** K.-U. Lee, S. Kim, J. T. Park, K. H. Choi

**Affiliations:** 1 Department of Psychiatry, Uijeongbu St. Mary’s Hospital, College of Medicine, The Catholic University of Korea; 2 Department of Psychiatry, Yeouido St. Mary’s Hospital, College of Medicine, The Catholic University of Korea; 3 Department of Emergency Medicine, Uijeongbu St. Mary’s Hospital, College of Medicine, The Catholic University of Korea, Seoul, Korea, Republic Of

## Abstract

**Introduction:**

Non-suicidal self-injury (NSSI) and suicide attempts (SA) represent overlapping yet clinically distinct forms of self-harm. While both behaviors share common psychological underpinnings such as emotional dysregulation and interpersonal difficulties, their intent, lethality, and clinical implications differ substantially. Understanding these differences is particularly critical in emergency department (ED) settings, where clinicians must rapidly assess risk and determine appropriate interventions.

**Objectives:**

This study aimed to delineate their unique risk, rescue, and psychopathology profiles among patients presenting to the emergency department (ED).

**Methods:**

We analyzed 356 ED patients with self-harm, categorizing them into NSSI (n = 61) and SA (n = 295) groups based on behavioral intent. Comprehensive comparisons were made across demographic, clinical, psychopathological, and situational (risk/rescue) variables.

**Results:**

The NSSI group was significantly younger, more often female, employed, and single (all *p* < 0.05). While both groups reported emotional distress, the SA group exhibited more severe depression, hopelessness, and comorbid medical illness (all *p* < 0.001), alongside higher prevalence of persistent suicidal ideation, planned acts, and use of high-lethality methods. In contrast, the NSSI group reported more frequent, impulsive episodes of self-injury—primarily by cutting—with minimal planning, no suicide notes, and low medical lethality (all *p* < 0.01). Interpersonal stress was the dominant precipitant for NSSI, whereas SA was more often associated with financial or interpersonal loss. Critically, the NSSI group demonstrated significantly lower suicide risk and medical severity (*p* < 0.001) but higher rescue potential, including more frequent overt help-seeking and better outcomes (all *p* < 0.01).

**Conclusions:**

NSSI and SA in the ED setting have distinct risk, rescue, and psychopathology profiles. NSSI is characterized by younger, more socially connected individuals engaging in impulsive, low-lethality acts with higher rescue potential, while SA involves older patients with more severe psychopathology, planned high-lethality behaviors, and poorer outcomes. Recognizing these differences is essential for accurate risk assessment and tailored intervention in emergency and acute care settings.

**Disclosure of Interest:**

None Declared

